# Parasites and Infection: Strategies to Control, Diagnose, and Treat Parasitic Diseases

**DOI:** 10.3390/microorganisms13061254

**Published:** 2025-05-29

**Authors:** Erica S. Martins-Duarte

**Affiliations:** Departamento de Parasitologia, Instituto de Ciências Biológicas, Universidade Federal de Minas Gerais, Belo Horizonte 31270-901, MG, Brazil; ericamduarte@ufmg.br

This Special Issue features thirteen publications on the basic biology, genetics, novel diagnostic tools and treatments, control strategies, disease epidemiology, and pathogenesis of medically significant protozoan and helminth parasites. Largely neglected, parasitic diseases contribute to high global morbimortality, particularly in communities lacking adequate sanitation, clean water, and healthcare. Consequently, reduced human work capacity and quality of life are prevalent in endemic areas. Such infections are frequently linked with malnourishment, iron deficiency, anemia, and compromised physical and intellectual development, along with other organic chronic conditions that impede regional growth [1].

However, fighting parasitic diseases presents considerable challenges, as local risk factors are constantly changing and differ widely according to each region’s unique environment, ecology, climate, culture, and socioeconomic conditions [2]. In addition, a critical hurdle for eradication in endemic areas is the lack of fast, sensitive, and specific diagnostic tools, effective treatments, and vaccines for most parasitic diseases. Therefore, continuous efforts are essential to thoroughly investigate parasite biology, local genetic variations, modes of transmission, main reservoirs, host immune responses, disease pathogenesis, new treatments, and drug resistance patterns to establish effective and sustainable prevention and control programs.

Helminth infections are common parasitoses in low- and middle-income countries. Among these, *Taenia solium* (pork tapeworm) infection is one of the most prevalent and endemic in sub-Saharan Africa, Asia, Latin America, and the Caribbean regions [3]. Transmission can occur by ingesting larvae in undercooked pork or eggs through the fecal–oral route, leading to various diseases. The ingestion of eggs, whether through self-contamination or the consumption of contaminated food or water, causes neurocysticercosis, a disease that can cause serious encephalic complications [4]. To better understand how oxidative stress contributes to inflammation and the pathophysiology of the disease, Generoso et al. [4] used a rat model that reproduces human extraparenchymal neurocysticercosis, a form of the disease less responsive to treatment and more prone to complications [4]. The obtained results showed a positive correlation between the presence of oxidative stress markers and inflammation intensity. These results encourage future efforts to monitor oxidative stress status during disease as a guide for better clinical intervention decisions for infected patients [5]. Another common helminth infecting humans is *Strongyloides stercoralis*, which causes high morbidity and possibly infects 600 million people globally [6]. It is an intestinal worm, and transmission occurs through skin penetration by larvae that contaminate the environment via shedding in the feces of infected individuals or animals. Based on previous reports of the potential of *Bacillus thuringiensis* crystal protein toxins against *S. stercoralis* and other gastrointestinal helminths, Pommare et al. (2024; [7]) conducted a pilot analysis using MALDi-TOF and a laboratory genomic pipeline study in stools of *S. stercoralis* infected and non-infected dogs to identify the genes involved in the synthesis of *B. thuringiensis* crystal toxins with possible larvicidal effect [7].

Intestinal parasitic diseases are globally distributed. However, they are most prevalent in developing countries, such as sub-Saharan Africa (SSA), Asia, Latin America, and the Caribbean [8]. These infections are commonly associated with malnourishment, iron deficiency, and anemia in children. For that reason, Cuna et al. [9] investigated whether intestinal parasites were the cause of the high anemia incidence in schoolchildren from a rural area in Bolivia. Although the authors did not find a correlation between anemia and intestinal parasite infections, a high rate of intestinal protozoan and helminths was found in those children. The most prevalent were the protozoans *Entamoeba coli* (48.9%) and *Blastocystis hominis* (40.2%). Still, other parasites of fecal–oral route transmission, such as *Ascaris lumbricoides*, *Giardia intestinalis*, and *Entamoeba histolytica*, were also found, suggesting precarious local sanitary structures [9].

The protozoan *Blastocystis* sp. is a common human intestinal parasite infecting humans and animals worldwide through the fecal–oral route. Usually, infection by this parasite is asymptomatic. However, some patients develop gastrointestinal symptoms, raising questions about whether specific genetic subtypes of the parasite are more likely to cause symptoms [10]. Matovelle et al. [11] explored the subtype diversity in fecal samples obtained from hospitalized patients exhibiting gastrointestinal symptoms from Northern Spain. They found two predominant (ST2 and ST3) and two minor (ST1 and ST4) subtypes. All four of these had been previously identified in patients with gastrointestinal symptoms. Mixed infections with different subtypes of *Blastocystis* were also found. The study of subtype distribution in humans and animals across different countries, along with their phylogenetic analysis and genetic diversity assessment, can help identify the primary sources of parasite transmission and elucidate differences in pathogenicity observed in some patients [11].

*Cryptosporidium* sp. is one of the world’s most frequent causes of diarrhea in humans and animals. Cryptosporidiosis has both anthroponotic and zoonotic spread, and transmission occurs through the fecal–oral route by consuming water or food contaminated with the feces of infected humans or animals [12]. In several countries, it is highly prevalent in children younger than 2–5 years old, and it is recognized as life-threatening for immunosuppressed individuals such as AIDS patients [13]. Diarrhea by *Cryptosporidium* species is also the main cause of mortality in neonatal calves, leading to substantial economic losses in dairy farms [14]. Two works published in this Special Issue examined the incidence of this parasite in animals and humans. In a systematic review and meta-analysis, Tawana et al. [15] showed a high infection rate of *Cryptosporidium* spp. from 1980 to 2020 in South Africa, with *Cryptosporidium parvum* being the prevalent species in both animals and humans. Among humans, HIV+ individuals showed greater exposure to *Cryptosporidium* than HIV- individuals, and higher infection rates were observed in regions where sanitary conditions are poorer. However, they also observed a decline in *Cryptosporidium* spp. infection prevalence from 2011 to 2020 compared to 2001–2010, indicating a possible improvement in local sanitary conditions, medication access, and good animal practice [15]. In another study, Kaduková et al. [16] demonstrated that in calves up to five weeks old in dairy farms in Eastern Slovakia, *C. parvum* is also the main infecting species of *Cryptosporidium*. Genotypic analysis showed that the subtype IIaA17G1R1 was the most prevalent in these calves. Considering that this subtype has already been reported in humans and that calves are the main reservoir of *C. parvum*, these results highlight the importance of preventive actions to reduce infection in those animals, environmental contamination, and the zoonotic spread of the parasite to humans [16].

Costa et al. [17] focused on the genetic diversity of *Toxoplasma gondii* by analyzing the alleles of five virulence factors. Their study examined 103 atypical strains of *T. gondii* isolated from humans, domestic, and wild animals from four different Brazilian States. The results showed a positive relation between the allele 4 of ROP18 and increased virulence and mortality in mice. These findings offer new perspectives for understanding how atypical virulence markers of *T. gondii* modulate the immune system in mice or humans. Further research in this direction could elucidate the higher virulence of atypical strains in mice and their connection to more severe toxoplasmosis outcomes observed Brazil [17]. 

*T. gondii* is an important, globally distributed protozoonosis commonly related to sequelae in congenitally infected newborns, ocular lesions, and encephalitis in immunosuppressed individuals [18]. Serological inquiry for this parasite infection is mandatory during early pregnancy to prevent congenital transmission [19]. In a systematic review and meta-analysis, Ribeiro et al. [20] determined the effectiveness of the main treatments and current treatment protocols for gestational and congenital toxoplasmosis, drawing on 56 studies from 16 countries. Their results demonstrated that treating acutely infected pregnant individuals with either spiramycin or triple therapy (sulfadiazine + pyrimethamine + folinic acid) reduces the risk of neonatal vertical infection and sequelae. These findings show that the serological monitoring of susceptible individuals during pregnancy, combined with appropriate treatment, has a good prognosis for reducing congenital toxoplasmosis [20].

The search for novel therapies for toxoplasmosis was the focus of the work of Costa et al. [21]. They employed a drug repositioning strategy to screen 160 drugs or drug-like compounds from the Medicines for Malaria Venture COVID Box to identify against *T. gondii*. A total of 23 molecules inhibited the tachyzoite forms of *T. gondii* in vitro by more than 70% at 1 µM after seven days of treatment. For two of them (apilimod and midostaurin), this was the first reported activity against this parasite. Morphological and ultrastructural analysis by transmission electron microscopy and fluorescence microscopy of tachyzoites treated with nine compounds showed that they all induced alterations in the parasite organelles and cell division [21].

Santos et al. [22] sought to understand the role of SAG2A in the *T. gondii* immune response by the peritoneal exudate cells (PECs) of susceptible and resistant mice. SAG2A is an immunodominant antigen expressed on the surface of the tachyzoite stage and, for that reason, is a promising candidate for vaccine and diagnostic development [23,24]. The results showed that recombinant SAG2A differentially modulates the immune response in the PECs of susceptible and resistant mice. PECs from susceptible mice were more sensitive to modulation by rSAG2A and showed lower parasitism than PECs from resistant mice. These results indicate that developing vaccines and new diagnostic tools for toxoplasmosis requires a better comprehension of the immune response variations from different hosts to *T. gondii* antigens [22].

The immune factors associated with the congenital transmission of the protozoan *Trypanosoma cruzi* were investigated by Herrera Choque et al. [25]. Chagas disease is an endemic zoonosis in South America, associated with poverty in rural areas and precarious housing conditions. It is estimated that *T. cruzi* causes the death of 10,000 individuals worldwide every year [26]. This parasite is primarily transmitted through contact with the feces or urine of blood-sucking triatomine bugs. However, congenital transmission occurs in around 10% of chronically infected pregnant individuals and poses a threat to disease eradication, as it can sustain transmission over time [27]. The immune and genetic factors involved in congenital transmission, however, remain unclear. The authors analyzed IFN-gamma concentration in maternal, placenta, and cord blood samples from both uninfected and infected mothers and their newborns. Higher levels of this cytokine were found in infected, non-transmitting mothers compared to transmitting ones, suggesting a possible role of IFN-gamma in controlling parasite infection and proliferation in the placenta and, consequently, in preventing fetal transmission [25].

Another highly relevant zoonotic disease is visceral leishmaniosis, caused by the *Leishmania infantum* species, especially in Brazil, which accounts for 97% of the total cases in America [28]. In endemic urban areas, dogs serve as the main reservoir for the parasite. Therefore, faster and more accurate diagnosis of infection in these animals is essential to enable timely treatment or, when necessary, euthanasia to prevent further transmission [29]. However, the protocols and diagnostic tools currently available in Brazil have limitations regarding sensitivity and specificity, hindering the detection of early seropositive or asymptomatic infected dogs [30]. To address this gap, Moreira et al. [31] used immunoinformatic tools to discover new peptides with higher sensitivity and specificity for detecting canine visceral leishmaniosis in asymptomatic dogs by ELISA assays. Two peptides were selected and tested, both individually and in combination. The combination of peptides showed 100% specificity in detecting infection in asymptomatic dogs. Additionally, a high specificity (90%) was observed in distinguishing infections caused by other pathogens or in vaccinated dogs. These data offer new possibilities for improving the management of canine visceral leishmaniosis in endemic areas [31].

Lastly, the review of Muh et al. [32] summarized recent findings on the biology, natural hosts and vectors, epidemiology, clinical presentation, and management of the simian malaria parasite *Plasmodium cynomolgi*, an emerging zoonotic pathogen in Southeastern Asia. *P. cynomolgi* was first reported in 2011, in a patient from the east coast of Peninsular Malaysia. Since then, the number of reported cases has increased throughout the region. However, to date, there has been no evidence that *P. cynomolgi* is naturally transmitted from one human to another; instead, proximity to natural reservoir hosts (macaque monkeys) remains the main risk factor for contracting *P. cynomolgi* malaria. The authors highlighted that extensive deforestation has significantly impacted agriculture and human settlements, likely bringing mosquito vectors into closer contact with both humans and reservoir hosts. Further research into the primary vectors, reservoirs, and disease manifestations in humans is essential to better understand this type of zoonotic malaria and to prevent potential, yet unknown, risks to human health [32].

Of the 11 parasites discussed above, 8 are zoonotic—an unsurprising fact given that approximately 60% of infectious diseases are of zoonotic origin, causing millions of deaths annually [33]. Changes in the natural ecosystems, biodiversity, and climate contribute to shifts in the distribution of pathogens, vectors, and hosts. As previously mentioned, population growth over the last and this century has led to the establishment of human settlements in deforested areas, bringing humans closer to wildlife, where the majority of emergent zoonosis originates [34,35,36]. The risk of zoonotic disease introduction or (re)-emergence is even more critical for the impoverished population, who are already burdened by persistent neglected zoonotic diseases and the most vulnerable to the risk of new zoonotic pathogens [37]. The adoption of a One Health strategy—which seeks to balance human health through investments in food/nutritional security, access to clean water, healthcare services, enhanced diagnostic tools and treatment, and improved housing and education, alongside sustainable animal welfare and agriculture, ecosystem protection, and biodiversity restoration—is crucial for eradicating the existing and preventing the (re)-emergence of parasitic diseases.
